# The risk of NTM pulmonary infection associated with trace metal exposure from public distribution system water in the United States

**DOI:** 10.1038/s41370-025-00807-w

**Published:** 2025-11-16

**Authors:** Ettie M. Lipner, Collin Powell, Stephen Nelson, Jonathan R. Fintzi, Joshua P. French, Rachel A. Mercaldo, Joseph O. Falkinham, D. Rebecca Prevots

**Affiliations:** 1 Epidemiology and Population Studies Section, National Institute of Allergy and Infectious Diseases, National Institutes of Health, Bethesda, MD USA; 2 Department of Geology and Geophysics, University of Utah, Salt Lake City, UT USA; 3 Office of Biostatistics Research, National Institute of Allergy and Infectious Diseases, National Institutes of Health, Bethesda, MD USA; 4 Department of Mathematical and Statistical Sciences, University of Colorado Denver, Denver, CO USA; 5 Department of Biological Sciences, Virginia Tech, Blacksburg, VA USA

**Keywords:** Environmental epidemiology, NTM, trace metals, pulmonary, drinking water

## Abstract

**Background:**

The prevalence of nontuberculous mycobacterial (NTM) pulmonary infection (NTM PI) varies geographically in the United States (US). Previous studies indicate that trace metals in environmental source water increase NTM PI risk.

**Objective:**

To examine the effect of trace metals, chromium (Cr), cobalt (Co), molybdenum (Mo), strontium (Sr), and vanadium (V) in public distribution system water on NTM PI risk among susceptible persons in the US.

**Methods:**

We studied NTM PI risk as a function of trace metal exposure from public distribution system water in two US patient populations: (1) The Cystic Fibrosis Foundation Patient Registry. (2) The Centers for Medicare and Medicaid Services. Patient data were extracted for the period 2010–2019. We obtained data on trace metal concentrations from the US Environmental Protection Agency, Third Unregulated Contaminant Monitoring Rule dataset. We used logistic and negative binomial generalized linear models to estimate NTM PI risk as a function of trace metal exposure in treated drinking water at the county level. Our models were adjusted for patient demographics, source water type, climate variables, and the type of disinfectant used.

**Results:**

Our cystic fibrosis (CF) population comprised 14,251 persons with CF, including 4020 NTM cases and 10,231 controls. Our Medicare population comprised 27,146,753 beneficiaries, including 84,075 NTM cases. In the Medicare analysis, V was associated with increased NTM PI risk in the Midwest, South, and West, while Mo increased NTM PI risk in the West. In the CF analysis, V increased NTM PI risk in the South, while Mo was associated with increased NTM PI risk in the West. In both the CF and Medicare populations, using chloramine as a disinfectant significantly increased NTM PI risk.

**SIGNIFICANCE:**

Mo and V in treated water was associated with increased risk among persons susceptible to NTM PI. The effect of trace metals varies by geographic region

**Impact:**

- The incidence and prevalence of NTM, a waterborne pulmonary infection, have been increasing in the US and have been shown to vary geographically. Our study has shown that trace metals, specifically molybdenum and vanadium, in treated drinking water significantly increase the risk of NTM pulmonary infection across the US. Our study also shows that the effect of trace metals varies by geographic region. Our research may lead to the monitoring of trace metals in drinking water as well as in susceptible populations, and ultimately, may be incorporated into a framework for NTM prevention efforts.

## Introduction

Nontuberculous mycobacteria (NTM) pulmonary infection (NTM PI) has become an increasingly prevalent waterborne pulmonary infection in the United States (US) [1, 2]. The incidence of NTM PI has been increasing in the US in both the cystic fibrosis (CF) and non-CF populations [3, 4] and is increasingly becoming a public health concern [2]. Therefore, identifying risk factors for exposure is central to prevention efforts.

NTM have been isolated throughout the water supply system, including natural source water [5, 6], water treatment facilities [5, 6], water distribution systems [5–7], premise plumbing [8, 9], potable water taps [5, 6, 10], and showerheads [11, 12]. NTM infections have been found to be the leading cause of drinking water-associated illnesses, emergency department visits, and hospitalizations [13]. Though exposure to NTM is common, the environmental distribution of NTM and the prevalence of NTM PI vary geographically in the US [11, 14–19]. These geographic differences are not explained by host-related factors and are likely due to variation in regional environmental conditions. Specific water-related factors favoring NTM growth and persistence likely increase the risk of exposure in particular environments.

In recent studies, we examined the relationship of NTM PI and water-quality constituents in environmental water sources that supply public water systems. These studies, conducted in three US states (Colorado, Hawai’i, and Oregon) [20–23] and at a subnational level across eleven US states [24], demonstrated that the trace metals molybdenum (Mo) and vanadium (V) in surface water and groundwater were associated with increased risk of NTM PI in both CF and non-CF populations. This evidence suggests that trace metal exposure from natural water sources may contribute to the geographic variation of NTM PI.

To estimate the effect of trace metal exposure from treated water in public distribution systems, we obtained US Environmental Protection Agency (US EPA) data on trace metal measurements from public water systems (PWSs) collected under the Third Unregulated Contaminant Monitoring Rule (UCMR 3) [25]. The US EPA uses the Unregulated Contaminant Monitoring Rule (UCMR) to collect data on contaminants that are suspected to be present in drinking water and do not have health-based standards set under the Safe Drinking Water Act (SDWA) [25]. We associated data from the Third Unregulated Contaminant Monitoring Rule (UCMR 3) with national CF patient data extracted from the Cystic Fibrosis Foundation Patient Registry (CFFPR) [26] and from the Centers for Medicare and Medicaid Services (CMS) [27]. Here, we describe two population-based analyses to investigate the relationship between trace metals in treated water and the risk of NTM PI among susceptible persons in the US while controlling for patient demographic variables and water disinfectants, as well as environmental variables, including climate variables and source water type.

## Materials and methods

### Data collection

#### Patient data

We obtained national NTM data from two sources: the CFFPR [26] and CMS [27]. Patient data from both sources were extracted for the study period 2010-2019.

The CFFPR includes data on >90% of persons with cystic fibrosis (pwCF) residing in the US. We included data on patients ≥ 12 years with a US zip code. We obtained data related to patient zip code, NTM pulmonary cultures, and patient demographics. Incident cases were defined as pwCF who had at least one positive NTM pulmonary culture preceded by two negative cultures during the study period and who lived in the same county for at least one year prior to their first positive culture. Controls were defined as pwCF who had at least two negative pulmonary cultures within a single county over a period of at least two years and had no history of positive cultures. Hereafter, we refer to this as the ‘CF analysis’. We also obtained patient data from the Medicare Part B claims dataset provided by CMS. Medicare Part B incident NTM cases were defined as Medicare beneficiaries aged >= 65 years who had (1) at least one claim associated with either ICD-9 031.0 (pulmonary diseases due to other mycobacteria) or ICD-10 A31.0 (pulmonary mycobacterial infections (other than tuberculosis)), (2) no prior claims associated with ICD-9 031.0 or ICD-10 A31.0 claims in the 24 months prior to the date of the incident claim, and (3) had a single reported county-level Federal Information Processing Standards (FIPS) code of residence for 24 months prior to the first claim. Hereafter, we refer to this as the ‘Medicare analysis’.

This study was classified as nonhuman subjects research by the National Institutes of Health, Office of Human Subjects Research Protection, because patient data were de-identified, and investigators could not link back to identifiable data.

#### Water-quality data

We obtained trace metal data from the US EPA UCMR 3 dataset [25]. The UCMR 3 required monitoring for 30 unregulated contaminants by public water systems (PWSs) between January 2013 through December 2015. Included in this dataset were all PWSs serving more than 10,000 people and 800 representative PWSs serving 10,000 or fewer people from across the fifty US states and the District of Columbia. Sampling points used in data collection occurred at entry points to the distribution system and at the distribution system's maximum residence time, indicating the point at which treated water has been in the distribution system for the maximum time.

**Fig. 1:**
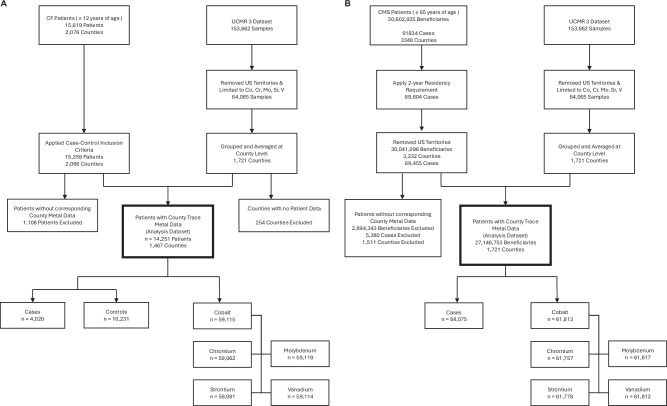
Flow diagrams for cystic fibrosis and Medicare analyses. **A** Identification of cystic fibrosis patients and UCMR 3 trace metal data included in analysis. **B** Identification of Medicare patients and UCMR 3 trace metal data included in analysis.

We accessed the UCMR 3 data on 3 January 2023. While the majority of PWSs reported their service area zip codes, if a given PWS did not report the latitude/longitude coordinates or at least one zip code, their associated water data were eliminated from our dataset. Our dataset included 4915 PWSs with trace metal data available and at least one zip code or latitude/longitude coordinates reported. We geocoded the zip code or latitude/longitude coordinates of each PWS to their respective county using the United States Postal Service (USPS) zip code database. We restricted the data to include only the five trace metals that were measured in the UCMR 3: chromium (Cr), Cobalt (Co), Mo, Strontium (Sr), and V (Fig. 1). Trace metal county-level statistics by region are presented in Table 1. Supplementary Table 1 shows the correlation matrix for Cr, Co, Mo, Sr, and V. Data were cleaned and analyzed using R (version 4.2.1), and R packages **MASS** [28], **corrplot** [29], **stats** [30], **effects** [31, 32], **car** [31]. The cleaned dataset used in this analysis required identifying and reformatting zip codes or latitude/longitude coordinate data entry errors. Our data cleaning steps are detailed in Appendix 1.

**Table 1:** Distribution of trace metals in PWSs stratified by U.S. regions, 2013-2015.

	Study Region	Number of PWSs	Number of observations	Arithmetic Mean Concentration µg/L	50^th^ percentile µg/L	75^th^ Percentile µg/L	90^th^ Percentile µg/L	95^th^ percentile µg/L	US EPA MRL µg/L	At or above MRL n (%)
CHROMIUM	Midwest	1148	9836	0.43	0.20	0.40	0.94	1.66	0.20	5302 (54%)
	Northeast	808	10,684	0.34	0.20	0.35	0.60	0.84	0.20	5596 (51%)
	South	1868	22,315	0.54	0.14	0.26	0.54	1.23	0.20	7624 (34%)
	West	1023	18,971	1.84	0.40	1.70	4.60	8.20	0.20	12667 (67%)
COBALT	Midwest	1148	9842	0.73	0.71	0.71	0.71	0.71	1.00	73 (0.7%)
	Northeast	808	10,712	0.91	0.71	0.71	0.71	0.71	1.00	400 (3.7%)
	South	1868	22,326	0.73	0.71	0.71	0.71	0.71	1.00	250 (1.1%)
	West	1023	18,982	0.72	0.71	0.71	0.71	0.71	1.00	109 (0.6%)
MOLYBDENUM	Midwest	1148	9842	2.45	1.10	2.40	5.00	7.90	1.00	5761 (59%)
	Northeast	808	10,712	0.94	0.71	0.71	1.00	1.50	1.00	1113 (10%)
	South	1868	22,328	1.72	0.71	1.30	2.82	5.50	1.00	6790 (30%)
	West	1023	18,984	3.01	1.53	3.60	5.80	8.90	1.00	11657 (61%)
STRONTIUM	Midwest	1148	9838	548	133	309	852	1446	0.30	9808 (99.7%)
	Northeast	808	10,703	159	84	160	283	488	0.30	10675 (99.7%)
	South	1868	22,321	324	120	325	714	1200	0.30	22291 (99.9%)
	West	1023	18,965	403	270	520	910	1100	0.30	18948 (99.9%)
VANADIUM	Midwest	1148	9842	0.71	0.20	0.48	1.50	3.10	0.20	5167 (53%)
	Northeast	808	10,712	0.39	0.14	0.28	0.75	1.40	0.20	3410 (32%)
	South	1868	22,327	1.27	0.21	0.68	2.70	5.07	0.20	11651 (52%)
	West	1023	18,980	6.59	2.80	8.50	19.00	26.00	0.20	16622 (88%)

We extracted data on source water type as reported by each PWS, which included surface water, groundwater, groundwater under the influence of surface water, and mixed water. Any county with all PWSs reporting the use of multiple water types was designated as mixed water use in our analysis. We also extracted data on the use of disinfectants reported by each PWS. We created four categories of disinfectants: Chlorine, Chloramine, Hypochlorite, and Other. The “Other” grouping was comprised of categories termed “chlorine dioxide”, “ozone”, “ultraviolet light”, “no disinfectant used”, and “all other types of disinfectants”, each of which was reported by only a small number of PWSs. Since most PWSs reported the use of multiple disinfectants, we used this variable in our analysis as follows: a given county was assigned to one or more disinfectant groups (i.e., Chlorine, Chloramine, Hypochlorite, Other), if the PWSs within that given county reported usage. Therefore, disinfectant categories were represented as present or absent for a given county.

Trace metal concentrations below the Minimum Reporting Level (MRL) were replaced by the MRL divided by the square root of two (this method was suggested in a personal communication with a United States Geological Survey (USGS) scientist, as well as having been used in previous studies [33]). For Cr, Co, Mo, Sr, and V, these MRL values were 0.141, 0.707, 0.707, 0.212, and 0.141 µg/L, respectively. The percent of trace metals that exceeded the MRL in each US region is shown in Table 1. We added 1 to individual trace metal measurements and then applied the natural log transformation to each value, so the transformed values were always greater than zero. We then aggregated all water samples by county and calculated the mean (average) trace metal measurement for each county. We next standardized the mean log concentrations to have a mean of 0 and a standard deviation of 1 at the regional level (Midwest, Northeast, South, West). Every patient was assigned the mean value (transformed and standardized) of each trace metal for their respective county of residence.

**Fig. 2:**
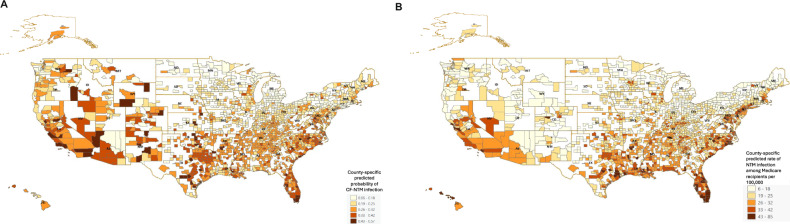
Estimated NTM infection risk by county. **A** County-level NTM risk among persons with CF. Grey lines represent county line boundaries, orange lines represent state line boundaries in the US. State abbreviations are printed in black. White regions represent counties without patient and/or water data. **B** County-level NTM risk among Medicare recipients. Grey lines represent county line boundaries, orange lines represent state line boundaries in the US. State abbreviations are printed in black. White regions represent counties without patient and/or water data.

Our final datasets excluded counties without trace metal data and included only counties that had patient data with corresponding trace metal data. In the CF analysis, counties with at least one case or one control in residence were included, representing 1467 counties, or 46.7% of all US counties. In the Medicare analysis, counties with at least 1 beneficiary in residence were included, representing 1721 counties, or 54.7% of all US counties. We obtained measurements for Cr, Co, Mo, Sr, and V from PWSs across the US (sample sizes are shown in Fig. 1). Counties with both patient and trace metal data are shown in Fig. 2A, B. Maps were generated using ArcGIS Pro (Environmental Systems Research Institute (Esri). 2022. ArcGIS Pro 3.0.0. Redlands, CA).

#### Climate data

To control for climate factors that influence risk, we selected climate predictors that are also associated with our exposures of interest. We obtained data on precipitation and floods for the study period, 2010–2019, from Weather Source, LLC. Precipitation was calculated as the mean of total yearly precipitation by county. Floods were the total number of Flood NWS Warnings for each county over the study period.

### Statistical analysis

#### Generalized Linear Models

We used generalized linear models (GLMs) to model NTM PI risk as a function of trace metal exposure in distribution system water. County-level standardized mean values of each trace metal were included. We constructed two models, one for the CF analysis and one for the Medicare analysis, that each included Cr, Co, Mo, Sr, and V as explanatory variables. The correlation matrix for these trace metals is shown in the Supplement (Supplementary Table 1). Demographic variables (age, sex, median income), climate variables (precipitation, flood warnings), water disinfectants (chlorine, chloramine, hypochlorite, other disinfectants), and source water type (surface water, groundwater, mixed water) were also included as covariates in each model. To account for interaction between Mo and V and between precipitation and floods, we included interaction terms for Mo and V and for precipitation and floods in the regression models. To account for regional influence on each of the variables, we included interaction terms between each model variable (demographic variables, source water type, climate variables, disinfectants used, and trace metals) and each of the four US regions: Midwest, Northeast, South, and West. We used the glht() procedure in the **multcomp** package [34] in R to adjust for multiple comparisons. We estimated the odds of NTM PI among pwCF and the rate of NTM PI among Medicare recipients given exposure to varying trace metal concentrations in PWSs across the US. We report an adjusted odds ratio in the CF analysis, an adjusted rate ratio in the Medicare analysis, 95% confidence interval (CI), and *p*-value for each model variable. Finally, we estimate the county-specific risk of NTM PI among pwCF and Medicare recipients based on trace metal exposure in PWSs (Fig. 2A, B).

## Results

### Study population characteristics

In the CF population, 14,251 pwCF who met our case or control definitions and had corresponding county-level trace metal data were included in our analysis. This population comprised 4,020 NTM cases and 10,231 culture-negative controls. In the Medicare population, 27,146,753 Medicare beneficiaries, who met our inclusion criteria and had corresponding county-level trace metal data, were included in our analysis. This included 84,075 Medicare patients who met our NTM case definition. Table 2 shows the demographic characteristics of CF cases and controls, as well as Medicare cases and beneficiaries residing in each US region (Midwest, Northeast, South, West).

**Table 2:** Descriptive statistics of cases and controls among a national cystic fibrosis (CF) population & cases and beneficiaries among a Medicare population.

Characteristic	CF NTM Cases (NTM culture-positive)		CF Controls (NTM culture-negative)		Medicare NTM Cases		Medicare Beneficiaries	
Count	All	4020	All	10,231	All	84,071	All	27,084,170
	West	974	West	2152	West	18,038	West	5,526,000
	Northeast	704	Northeast	2080	Northeast	16,601	Northeast	5,521,694
	South	1597	South	3493	South	36,844	South	10,472,013
	Midwest	745	Midwest	2506	Midwest	12,588	Midwest	5,564,463
**CF:** Age, yr, (Mean ± SD) **Medicare:** Age, % over 75, (County Mean ± SD)	All	28.5 ± 12.6	All	26.6 ± 12.0	All	60.6 ± 18.6	All	45.5 ± 3.78
	West	29.1 ± 12.7	West	27.2 ± 12.4	West	61.9 ± 17.6	West	42.8 ± 4.36
	Northeast	30.4 ± 13.3	Northeast	27.6 ± 12.2	Northeast	60.2 ± 13.9	Northeast	46.8 ± 3.06
	South	27.4 ± 12.3	South	25.5 ± 11.5	South	59.0 ± 18.6	South	44.8 ± 3.11
	Midwest	28.5 ± 12.0	Midwest	26.8 ± 12.2	Midwest	62.8 ± 20.2	Midwest	47.8 ± 3.68
**CF:** Female sex, *n* (%) **Medicare:** % Female sex, (County Mean ± SD)	All	1949 (48.5)	All	4859 (47.5)	All	68.6 ± 35.6	All	54.6 ± 2.06
	West	493 (50.6)	West	1029 (47.8)	West	69.9 ± 33.5	West	52.0 ± 2.08
	Northeast	343 (48.7)	Northeast	947 (45.5)	Northeast	69.0 ± 30.7	Northeast	55.1 ± 1.68
	South	768 (48.1)	South	1693 (48.5)	South	67.7 ± 35.6	South	55.0 ± 1.81
	Midwest	345 (46.3)	Midwest	1190 (47.5)	Midwest	69.2 ± 38.2	Midwest	54.8 ± 1.72
Median income* (per $1000)	All	53.2 ± 13.4	All	53.0 ± 13.6	All	77.7 ± 19.8	All	77.7 ± 19.8
	West	57.0 ± 11.7	West	56.1 ± 10.9	West	83.2 ± 22.1	West	83.2 ± 22.1
	Northeast	57.8 ± 15.0	Northeast	58.8 ± 15.6	Northeast	92.7 ± 25.0	Northeast	92.7 ± 25.0
	South	49.7 ± 13.3	South	49.4 ± 14.2	South	72.5 ± 18.3	South	72.5 ± 18.3
	Midwest	51.5 ± 11.1	Midwest	50.4 ± 10.4	Midwest	78.8 ± 14.4	Midwest	78.8 ± 14.4

^*^Median income for the CMS population was obtained from the American Community Survey^1^.

West: AK, AZ, CA, CO, HI, ID, MT, NV, NM, OR, UT, WA, WY.

Northeast: CT, ME, MA, NH, NJ, NY, PA, RI, VT.

South: AL, AR, DE, DC, FL, GA, KY, LA, MD, MS, NC, OK, SC, TN, TX, VA, WV.

Midwest: IL, IN, IA, KS, MI, MN, MO, NE, ND, OH, SD, WI.

### Generalized linear models

The interpretation of final models assumes that additional variables in the model remain fixed while the value of the variable being interpreted changes. For pwCF living in Southern counties, the odds of NTM infection increased by 27% (95% CI 1.05, 1.54) for every unit increase in, V and in Western counties, the odds of NTM infection increased by 32% (1.09, 1.61) for every unit increase in Mo (Table 3A). While we did not observe any statistically significant associations in other regions, the effect of V in the Midwest was borderline significant and, with a larger sample size may have achieved significance (Table 3A). For Medicare recipients living in Midwestern counties, every 1- standardized log unit increase (hereafter referred to as ‘unit increase’) in V in public water increased the incidence rate of NTM PI by 13% (1.02, 1.24) (Table 3B). In Southern counties, the incidence rate of NTM PI decreased by 9% (0.85, 0.97) for every unit increase in Cr. We also observed a significant interaction effect between V and Mo for counties in the South (Table 3B). The risk of NTM PI increased by 15% (1.06, 1.24) for every unit increase in V when the concentration of Mo was at its mean value in Southern counties (Table 3B) and increased by 18% (1.07, 1.30) for every unit increase in V when Mo was at its minimum value in Southern counties (Supplementary Table 2B). In Western countries, the incidence rate of NTM infection increased by 10% (1.01, 1.19) for every unit increase in V and 12% (1.01, 1.25) for every unit increase in Mo (Table 3B). We did not observe significant associations for any metals in the Northeast in either the CF or Medicare analyses.

**Table 3:** A) The odds ratio of NTM pulmonary infection among persons with cystic fibrosis in the United States for trace metals and water disinfectants. B) The rate ratio of NTM pulmonary infection among Medicare recipients in the United States for trace metals and water disinfectants.

Region	A.	NTM PI among CF patients^1^ OR (95% CI) *p*-value^2^	B.	NTM PI among Medicare recipients^1^ RR (95% CI) *p*-value2
Midwest	Chromium	1.06 (0.90,1.24) 0.872	Chromium	0.97 (0.91,1.04) 0.748
	Cobalt	0.98 (0.77,1.24) 0.998	Cobalt	1.05 (0.95,1.15) 0.643
	Molybdenum	0.96 (0.85,1.08) 0.859	Molybdenum	0.97 (0.91,1.02) 0.375
	Strontium	1.16 (0.96,1.41) 0.179	Strontium	0.96 (0.89,1.04) 0.639
	Vanadium	1.24 (0.98,1.57) 0.087	**Vanadium**	**1.13 (1.02,1.24) 0.007**
	Molybdenum: Vanadium	0.89 (0.73,1.08) 0.430	Molybdenum: Vanadium	0.99 (0.93,1.08) 1.000
	Chlorine	1.10 (0.76,1.59) 0.999	**Chlorine**	**1.18 (1.01,1.36) 0.024**
	Chloramine	1.02 (0.75,1.39) 1.000	**Chloramine**	**1.32 (1.11,1.57) 5.3×10**^**-5**^
	Hypochlorite	1.06 (0.77,1.45) 0.999	Hypochlorite	1.07 (0.92,1.26) 0.961
	Other disinfectants	1.28 (0.95,1.73) 0.211	Other disinfectants	0.87 (0.55,1.37) 0.999
Northeast	Chromium	1.33 (0.82,2.15) 0.448	Chromium	1.04 (0.69,1.55) 0.999
	Cobalt	0.97 (0.79,1.19) 0.993	Cobalt	0.98 (0.88,1.09) 0.986
	Molybdenum	0.77 (0.40,1.49) 0.796	Molybdenum	0.98 (0.71,1.36) 0.999
	Strontium	0.92 (0.72,1.19) 0.901	Strontium	1.01 (0.87,1.17) 0.999
	Vanadium	1.36 (0.79,2.33) 0.509	Vanadium	1.01 (0.67,1.52) 0.999
	Molybdenum: Vanadium	2.05 (0.52,8.17) 0.578	Molybdenum: Vanadium	1.67 (0.72,3.90) 0.418
	Chlorine	0.96 (0.66,1.37) 0.999	Chlorine	0.91 (0.73,1.15) 0.889
	Chloramine	1.45 (0.98,2.16) 0.083	Chloramine	1.10 (0.82,1.45) 0.999
	Hypochlorite	1.29 (0.78,2.14) 0.882	Hypochlorite	1.11 (0.88,1.40) 0.951
	Other disinfectants	0.97 (0.69,1.38) 1.000	Other disinfectants	0.84 (0.51,1.38) 0.997
South	Chromium	0.92 (0.80,1.06) 0.456	**Chromium**	**0.91 (0.85,0.97) 4.9 × 10**^**-4**^
	Cobalt	0.96 (0.88,1.05) 0.761	Cobalt	0.99 (0.96,1.02) 0.796
	Molybdenum	0.88 (0.76,1.03) 0.145	Molybdenum	1.06 (0.98,1.13) 0.173
	Strontium	1.02 (0.92,1.12) 0.992	Strontium	0.95 (0.91,1.00) 0.050
	**Vanadium**	**1.27 (1.05,1.54) 8.7×10**^**-3**^	**Vanadium**	**1.15 (1.06,1.24) 4.9 × 10**^**-5**^
	Molybdenum: Vanadium	0.90 (0.79,1.03) 0.194	**Molybdenum: Vanadium**	**0.89 (0.85,0.94) 3.0 × 10**^**-8**^
	Chlorine	0.94 (0.76,1.16) 0.999	Chlorine	0.94 (0.85,1.04) 0.644
	**Chloramine**	**1.35 (1.09,1.67) 5.4×10**^**-4**^	**Chloramine**	**1.39 (1.23,1.58) 3.7 × 10**^**-14**^
	Hypochlorite	1.01 (0.83,1.24) 1.000	Hypochlorite	1.09 (0.98,1.2) 0.266
	Other disinfectants	0.89 (0.72,1.11) 0.885	Other disinfectants	1.12 (0.78,1.62) 0.999
West	Chromium	1.04 (0.92,1.18) 0.894	Chromium	1.02 (0.95,1.10) 0.917
	Cobalt	0.69 (0.36,1.33) 0.489	Cobalt	0.84 (0.64,1.12) 0.436
	**Molybdenum**	**1.32 (1.09,1.61) 1.3×10**^**-3**^	**Molybdenum**	**1.12 (1.01,1.25) 0.034**
	Strontium	1.01 (0.79,1.29) 0.999	Strontium	0.98 (0.87,1.11) 0.996
	Vanadium	0.99 (0.85,1.17) 0.999	**Vanadium**	**1.10 (1.01,1.19) 0.017**
	Molybdenum: Vanadium	1.01 (0.89,1.15) 0.999	Molybdenum :Vanadium	0.95 (0.88,1.02) 0.253
	Chlorine	0.83 (0.62,1.12) 0.689	Chlorine	1.06 (0.87,1.30) 0.999
	Chloramine	0.92 (0.66,1.27) 0.999	Chloramine	1.13 (0.89,1.46) 0.922
	Hypochlorite	1.06 (0.61,1.83) 0.999	**Hypochlorite**	**1.24 (1.01,1.53) 0.027**
	Other disinfectants	1.12 (0.85,1.49) 0.984	Other disinfectants	0.98 (0.64,1.50) 1.000

Each row indicates the estimated effect estimate (odds ratio or rate ratio), the 95% confidence interval^1^ for the effect estimate, and the significance level of the effect estimate associated with a specific covariate. The effect estimate compares the odds or rate of NTM infection when the specific covariate increases from a fixed value to the fixed value plus 1 unit while the other covariates are held fixed.

^1^Source water type, sociodemographic variables, climate variables, and interaction term (between precipitation and floods) are also controlled for in the logistic model (A) and the negative binomial model (B). Full model results are available in the Supplementary Materials.

^2^Confidence intervals and p-values are adjusted for multiple comparisons.

Molybdenum :Vanadium indicates an interaction term.

Among disinfectant types, chloramine usage showed a significantly increased risk of NTM PI among Medicare patients in the Midwest and South. We observed an increase of 32% (1.11, 1.57) in the Midwest, and 39% (1.23, 1.58) in the South, where PWSs reported chloramine usage as a disinfectant compared with those PWSs who did not (Table 3B). We also observed an increase of 18% (1.01, 1.36) in the Midwest, where PWSs reported chlorine usage compared with PWSs that did not, and an increase of 24% (1.01, 1.53) in the West, where PWSs reported hypochlorite usage. In the CF analysis, we observed a 35% (1.09, 1.67) increase in the odds of NTM infection in the South, where PWSs reported chloramine usage as a disinfectant compared with those who did not report such usage (Table 3A).

We also observed that increasing precipitation in the Midwest increased risk of NTM PI in both populations. The number of flood warnings was not significantly associated with NTM PI. Supplementary Table 3A & 3B show results for all model covariates, including demographic variables, climate variables, interaction terms, and source water type. Supplementary Table 4 describes *Mycobacterium avium* Complex (MAC) and *Mycobacterium abscessus* specific modeling results for pwCF.

### Estimated county-level risk of NTM infection

We estimated the nationwide county-level risk of NTM infection among pwCF (Fig. 2A) and among Medicare recipients (Fig. 2B) based on our models (Table 3). For NTM PI among pwCF (Fig. 2A), we observed counties with the highest risk of infection in Western states, particularly in central and southern California, southern Nevada, southern Arizona, and throughout the Rocky Mountain states. We also observed a high risk of infection in certain counties in Kansas, and throughout the South, particularly in Florida, Texas, Oklahoma, Arkansas, Louisiana, and along the Eastern Seaboard. For NTM infection among Medicare recipients (Fig. 2B), counties with the highest risk of infection were located throughout the South, particularly in Florida, Texas, Oklahoma, Arkansas, Louisiana, Mississippi, and along the Eastern Seaboard, as well as in central and southern California, southern Nevada, and southern Arizona.

## Discussion

In this study, we reported significant associations between Mo and V in PWSs and NTM PI risk in the US. Moreover, similar associations were observed in two high-risk yet distinct populations. In both the CF and Medicare populations, Mo concentrations in the West and V concentrations in the South were strongly associated with NTM PI. For Medicare, we observed that V was also a significant risk factor for NTM PI in the Midwest and the West. In the South, we observed that Cr was protective for NTM PI. For CF, while the significant associations were limited to the South and West, V in the Midwest had a borderline risk effect, and with a larger sample size, may have achieved statistical significance, which would have been consistent with the Medicare results in this region.

Earlier work has identified hot spots of NTM across the US [19]. In particular, counties in Louisiana were identified as hot spots for NTM pulmonary disease [19]. This finding was consistent with other reports showing that NTM are found in high numbers in samples of peat-rich soils and acidic, brown water coastal swamps that are prevalent in Louisiana [16]. Epidemiologic studies of NTM PI risk in Colorado [20, 21], Hawai’i [23], Oregon [22], and a subnational analysis of the US found that NTM PI risk was positively associated with Mo and/or V concentrations in source water (i.e., streams, rivers, reservoirs, springs, groundwater) which supply public drinking water systems.

**Fig. 3:**
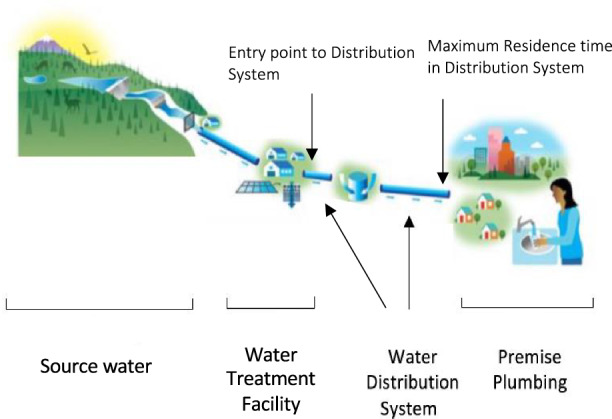
Public drinking water from source to tap. Bull Run Water Treatment. Water Quality Report. West Slope Water District. 2019; http:www.wswd.org/document_center/Water%20Quality/2019%20Water%20Quality%20Report.pdf. Accessed 25 April 2020.

In this study, we focused on public drinking water systems because the vast majority of the US population (86%) relies on public water supplies for their drinking water [35]. In the US, a population-based survey from the 1990s found that an estimated 87% of time was spent indoors [36], therefore, household water is an important source of exposure [8, 9, 37–40]. Drinking water, or ‘tap water’, originates from upstream bodies of water that are either surface water or groundwater, otherwise known as source water, which then becomes the water supply for PWSs. Between the source and the tap, distribution systems connect water treatment facilities to premise plumbing through miles of underground pipes, storage facilities, and pumps. In the UCMR 3 dataset, trace metals were measured at entry points to the distribution system as well as at the distribution system maximum residence time sampling locations (Fig. 3). Thus, we used distribution system water data, which directly represents the trace metal content of treated water that flows from household water taps.

Trace metals, Cr, Co, Mo, Sr, and V, are naturally occurring and are abundant throughout the earth’s crust [41]. These metals are considered trace elements because they are present in the environment at trace concentrations, as well as being essential and nonessential elements for human, animal, and plant life [42]. While natural environmental changes, such as rock weathering, soil erosion, and volcanic eruptions, contribute to the release of trace metals into our waterways, anthropogenic activities are also a major source of environmental contamination and human exposure to trace metals. Industrial activities, such as mine drainage, oil/gas and coal combustion, metal-based industrial production, and agricultural runoff, release large amounts of trace metals into the environment. The release of trace metals from industrial sources may occur in localized areas where trace metal concentrations become elevated and ultimately travel downstream to nearby waterways.

Region-specific characteristics of soil and water, as well as engineered water systems, including biofilms that form in hospital and public drinking water supplies, contribute to increased concentrations of NTM, leading to greater potential for NTM exposure and infection. Herein, we document that the distribution of NTM PI risk across US counties is non-uniform, distinctive, and unique.

To better understand potential environmental influences on NTM PI risk, we examined selected counties from Fig. 2A, B with respect to probable geogenic (factors that originate from geological sources) and anthropogenic V or Mo sources. Previously we focused on the naturally occurring effects of V and Mo on NTM PI risk [24]. Here, we focus our discussion on the anthropogenic sources of Mo and V with the caveat that it is sometimes difficult to separate geogenic from anthropogenic sources of metals.

### Case studies

**Fig. 4:**
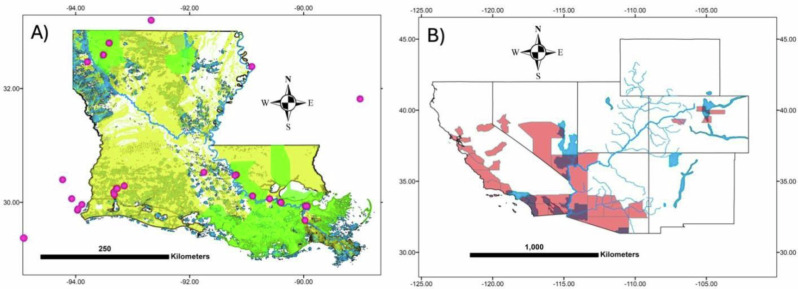
Case Studies. Selected high-risk counties in **A** Selected high-risk counties (parishes) in Louisiana are shown in solid green. Yellow patterns are mapped clay-rich bedrock and sediments (Horton, 2017) known or suspected to contain high transition metal contents. Blue polygons or patches are known oil fields and appear green where they coincide with clay-rich material. Large magenta dots are oil refineries, including several in nearby states. **B** A map of the Colorado River and major tributaries. Municipalities and unincorporated areas using Colorado River water are indicated in blue patterns (website below), along with high-risk counties in red, creating purple regions where they overlap. See text for discussion. https://coloradoriverbasin-lincolninstitute.hub.arcgis.com/. Accessed 22 Jan 2024. Horton JD, San Juan CA, Stoeser DB. The State Geologic Map Compilation (SGMC) geodatabase of the conterminous United States. Data Series 1052. Available from: https://pubs.usgs.gov/publication/ds1052.

The counties highlighted below represent the two highest categories of risk for NTM PI having elevated concentrations of V and Mo metals in their water sources. Two cases are examined as illustrated in Fig. 4A, B.

#### Louisiana & Oil Refineries

A cluster of high-risk counties for NTM was observed in southern Louisiana in the vicinity of the Mississippi River delta (Fig. 4A). Prominent near-surface geological features of the state are widespread clay deposits and a complex interconnected system of streams, wetlands, and lakes. Ho et al [43]. (2019) noted that groundwater discharge to the Gulf of Mexico locally influences V budgets, where releases of V and Mo were associated with reducing conditions [44, 45]. In addition, significant contamination of soils with V was reported due to flooding associated with Hurricane Katrina in 2007 [46].

Twenty-one oil refineries producing nearly half of the nation’s distillates are located in Louisiana [46]. Overall, more than 100 petrochemical plants are found along the Mississippi River between Baton Rouge and New Orleans [47]. V is known to occur in elevated concentrations in certain types of oil products and their distillates [48] and is emitted from steel mills, power plants, and oil refineries as well as through fossil fuel combustion, sewage sludge,e and fertilizers. Most of the high-risk counties for NTM PI are in the vicinity of these refineries. Mo releases are associated with mining and smelting, fossil fuel combustion, waste incineration, and fertilizers [49, 50]. The controls on V and Mo levels in domestic water supplies are less clear but likely involve both natural and anthropogenic sources.

#### Colorado River drainage & water transport

Lipner et al. [24] noted that water transferred from one surface drainage system to another area can confound relationships of geogenic sources to disease, in particular, water from the Colorado River. Colorado is a major producer of Mo, and mines along with their tailings and spoil rock occur within drainages contributing to the Colorado River, as well as draining into the South Platte. As such, there may be both geogenic and anthropogenic sources of Mo from the extractive industries of Colorado.

Here, we examine the case of water exports through water development projects. Instead of local geogenic or anthropogenic sources of V and Mo being important, imported sources of potable water may control the risk. Figure 4B illustrates the spatial relationship between high-risk counties and areas where Colorado River water is both exported and used within the basin.

The high-risk county (Yuma) in extreme southwest Arizona is adjacent to the Colorado River and has a major tributary (Gila River) that transects it. Yuma City, Arizona, for example, obtains its water from the Colorado River [51]. Las Vegas, Nevada, in Clark County imports the large majority of its water from the Colorado River [52] through a system of tunnels from Lake Mead [53], a large reservoir on the Colorado River. Elsewhere, water exported from the Colorado River basin is clearly associated with elevated risk in heavily populated southern California. Indeed, it is unclear how much of the hazard along the Arkansas River corridor is due to local geogenic sources, urbanization (Denver metropolitan area), and agriculture versus how much is due to exported Colorado River water.

### Trace metals and NTM infection hypotheses

We propose two hypotheses to explain the observed distribution of NTM PI risk and suggest potentially causal mechanisms.

### Exposure to Mo and V increase the abundance of NTM in public drinking water

We hypothesize that NTM numbers in public drinking water are influenced by the concentration and availability of metal ions. Specifically, water from counties with a high risk of NTM PI would also have high concentrations of Mo or V. Mo would serve as an essential metal because public drinking water is nutrient-poor and low in organic nitrogen but has available nitrate. MAC can use nitrate as a sole source of nitrogen (i.e., nitrate assimilation), due to the presence of the enzyme nitrate reductase, a Mo-dependent enzyme. We hypothesize that the enzyme may also use V to replace Mo. Nitrate reductase reduces nitrate to nitrite, and that pathway ultimately leads to the formation of ammonia, which is used to aminate fatty acids, producing amino acids used for protein synthesis [54]. The presence of Mo or V in public drinking water may provide the metal-cofactor for the use of nitrate as the sole nitrogen source.

If environmental source water in certain regions has elevated trace metal concentrations and PWSs draw water from that location, it will introduce a higher abundance of NTM into the water distribution system, thereby increasing the risk of infection. We hypothesize that environmental source water with a high abundance of NTM could initiate the presence of NTM in the water distribution system compared with PWSs drawing from source water with low NTM abundance. This effect may explain why certain counties have a greater infection risk than others.

Water distribution pipework may have little effect on the supplied drinking water trace metal concentrations. Smedley et al. [55] compared old and new houses, pre-flush and post-flush samples, tap water, and water samples from their respective source water in England and Wales, and found a negligible effect on the Mo concentrations of the water samples. As a result, the observed Mo concentrations in public drinking water systems are likely due to the concentrations of Mo in the source water. We did not find literature to assess the potential changes of V concentrations due to pipework materials.

### Exposure to Mo and V via water consumption increases human susceptibility to NTM PI

Mo and/or V may also influence host susceptibility by altering the ability of the human host to respond to or contain infection. A Korean study [56] reported that pulmonary NTM patients had significantly higher Mo concentrations in their blood serum compared with control patients. In a Sicilian study [57], authors examined trace metal concentrations in drinking water and in urine of residents living in basaltic volcanic regions (with naturally occurring elevated V concentrations) compared with non-volcanic regions. They reported that mean V concentrations in drinking water were significantly correlated with mean urinary V concentrations of residents in volcanic areas compared with non-volcanic areas. Thus, patients who live in regions with elevated Mo and/or V levels in their drinking water may be chronically exposed and could have increased susceptibility to infection and/or rapid progression of disease.

### Interaction effect

**Fig. 5:**
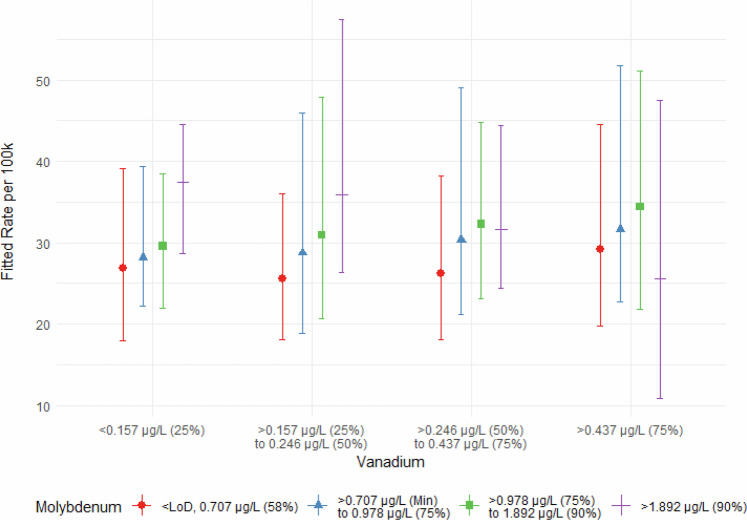
Vanadium and molybdenum interaction effect for NTM PI risk in the South. Lines on the plot represent fixed low, moderate, and high molybdenum concentrations in the distribution system water. The corresponding-colored regions represent confidence intervals for each line. The x-axis represents increasing vanadium concentrations in the distribution system water. The y-axis represents the expected number of NTM cases among Medicare recipients in the South.

Importantly in the Medicare analysis, a significant interaction effect between Mo and V was observed in the South, where at high fixed Mo concentrations (> 90^th^ percentile), the rate of NTM cases decreased as V concentrations increased. However, at lower fixed Mo concentrations (minimum molybdenum concentration to 90^th^ percentile), the rate of NTM cases increased as V concentrations increased (Supplementary Table 2B; Fig. 5). Importantly, out of 848 counties in the South, 491 counties had molybdenum values below the MRL and were therefore assigned the 0.707 μg/L value (Supplementary Table 2B). Previous literature has shown that NTM growth was stimulated by low concentrations of heavy metal salts, while growth was inhibited by high concentrations of some metals, and tolerated by high concentrations of other metals, including mercury and cadmium [58]. Although Mo and V were not among the metals examined in Falkinham et al. [58], we hypothesize, as described above, that in conditions where Mo is at low availability, NTM may use V in place of Mo as an essential co-factor for metabolism [59]. For this reason, we included an interaction term for Mo and V in our regression models, which proved to be statistically significant in the South of the Medicare analysis. No significant interaction effects were observed in the CF analysis. Further research is needed to more fully explore the relationship between Mo, V, and NTM PI risk.

### Disinfectant use

Chloramine use showed a consistent risk effect for NTM PI in multiple U.S. regions. These results are in line with the current literature. King et al. [6] reported that MAC detection frequencies were higher in chloramine-treated surface water than in chlorine-treated surface water. Donohue et al. [60] reported higher *M. avium* detection frequencies in chloramine-treated water than chlorine-treated water. Pfaller et al. [61] also reported that MAC was detected more frequently in surface water samples that were treated with chloramine than in those that were treated with chlorine. This association may be a manifestation of nitrification to produce nitrate in water treatment plants, where Mo (or potentially V as a substitute), as a cofactor of nitrate reductase, would then stimulate NTM numbers. While we also observed that chlorine use in the Midwest and hypochlorite use in the West were significant risk factors for NTM PI among Medicare recipients, the effect estimates were not as strong as those of chloramine use.

Our study had several limitations. The study population included patients over a longer time frame (2010–2019) than the years in which data collection for trace metals had occurred (2013–2015). However, when we analyzed the concentrations of trace metals by year, we did not find much fluctuation. Therefore, to maximize power, we used all available patient data and proceeded under the assumption that trace metal concentrations would remain fairly constant over the nine-year span. In addition, upon examining the raw data, we became aware that PWSs did not always report all the zip codes served. As a result, some counties may not have been included in our analysis because of a lack of trace metal data. However, rather than exclude disinfectants from our analysis, we retained the disinfectant variable in our models since it represents the real-world scenario of drinking water treatment. Supplementary Table 5 shows the results when disinfectant type was excluded from our analysis.

Our study also had several strengths. First, by using two high-risk populations for NTM PI, we had greater statistical power to identify exposures associated with NTM PI. Second, while previously we observed these associations using environmental source water, most people are not directly exposed to environmental water except through recreation. So, the question remained: would we observe these associations when using treated water from the distribution systems that serve residential homes, office buildings, hospitals, and industry? As a result, the trace metal variables in our models are measured from treated water, which people have direct exposure to in their homes, lending further support for a potentially causal relationship.

Another strength of our study comes from incorporating precipitation and flood data into our analysis. We controlled for these variables because they are likely to affect the trace metal content in the water supply, since greater precipitation and flooding can easily transport labile metals in soil and sediment into streams. Precipitation and floods have also been linked to increased risk of NTM PI [62, 63].

While our findings underscore a potential causal relationship between trace metals in the water supply and NTM PI risk, our work has raised important research questions. First and foremost, this study cannot differentiate between the influence of trace metals on bacterial growth and/or pathogenicity versus their influence on infection susceptibility and/or disease progression in the human host. Further, we have not examined a dose-response relationship between metals and infection. Environmental investigations of well and stream waters, urban runoff, irrigation return, and oil refinery emissions in selected high- and low-risk counties may permit estimation of the contribution of geogenic and anthropogenic sources to NTM infection. Finally, our research may lead to the monitoring of selected trace metals in both drinking water and susceptible populations, which could ultimately be incorporated into a framework for NTM prevention efforts.

## Supplementary information

Supplementary information

## Data Availability

The data that support the findings of this study are available from the Centers for Medicare and Medicaid Services and from the Cystic Fibrosis Foundation. Restrictions apply to the availability of these data, which were used under a Data Use Agreement for the current study, and so are not publicly available.
