# Supplementary material for: The risk of NTM pulmonary infection associated with trace metal exposure from public distribution system water in the United States
Source: J Expo Sci Environ Epidemiol. 2025 Nov 16;36(2):375–85. doi: 10.1038/s41370-025-00807-w (PMC12960223; doi:10.1038/s41370-025-00807-w)
Supplement: Supplementary file 1 — Supplementary information [file 41370_2025_807_MOESM1_ESM.docx]

Supplemental Tables

Supplemental Table 1. Trace metal correlation matrix

| **Trace metal** | Cr | Mo | V | Sr | Co |
| --- | --- | --- | --- | --- | --- |
| Chromium  (Cr) | 1.00 |  |  |  |  |
| Molybdenum  (Mo) | **0.16**** | 1.00 |  |  |  |
| Vanadium  (V) | **0.61**** | **0.28**** | 1.00 |  |  |
| Strontium (Sr) | **0.19**** | **0.50**** | **0.33**** | 1.00 |  |
| Cobalt (Co) | -0.02 | **-0.06*** | **-0.05** | **-0.08**** | 1.00 |

(bold: p<0.05, bold*: p<0.01, bold**: p<0.001)

Supplemental Table 2A. Comparison of odds ratio of NTM infection under

different Molybdenum and Vanadium scenarios in a CF population.

| Midwest | 25% Quantile Molybdenum (0.940 μg/L) | 50% Quantile Molybdenum (1.430 μg/L) | 75% Quantile Molybdenum (2.674 μg/L) | 90% Quantile Molybdenum (5.306 μg/L) |
| --- | --- | --- | --- | --- |
| Vanadium odds ratio  95% CI  p-value | 1.373  (0.94, 2.01)  0.180 | 1.296  (0.95, 1.76)  0.163 | 1.167  (0.91, 1.49)  0.506 | 1.017  (0.71, 1.45)  1.000 |
| Northeast | 25% Quantile Molybdenum (0.707 μg/L) | 50% Quantile Molybdenum (0.717 μg/L) | 75% Quantile Molybdenum (0.832 μg/L) | 90% Quantile Molybdenum (5.306 μg/L) |
| Vanadium odds ratio  95% CI  p-value | 1.227  (0.62, 2.44)  0.986 | 1.238  (0.63, 2.44)  0.981 | 1.367  (0.74, 2.52)  0.757 | 1.645  (0.84, 3.22)  0.300 |
| South | 25% Quantile Molybdenum (0.707 μg/L) | 50% Quantile Molybdenum (0.752 μg/L) | 75% Quantile Molybdenum (1.357 μg/L) | 90% Quantile Molybdenum (2.002 μg/L) |
| Vanadium odds ratio  95% CI  p-value | 1.324  (1.03, 1.70)  0.019 | 1.316  (1.03, 1.68)  0.019 | 1.234  (1.01, 1.51)  0.039 | 1.171  (0.96, 1.43)  0.215 |
| West | 25% Quantile Molybdenum (0.905 μg/L) | 50% Quantile Molybdenum (1.657 μg/L) | 75% Quantile Molybdenum (3.856 μg/L) | 90% Quantile Molybdenum (4.663 μg/L) |
| Vanadium odds ratio  95% CI  p-value | 0.989  (0.82, 1.20)  1.000 | 0.996  (0.84, 1.18)  1.000 | 1.009  (0.77, 1.32)  1.000 | 1.013  (0.75, 1.37)  1.000 |

The odds ratio compares the odds of NTM infection when Vanadium increases from a fixed value, $V_{0}$, to $V_{0}+1$ while Molybdenum is held fixed at a value, $M_{0}$. We computed the odds ratios under four fixed values of $M_{0}$. The fixed value of $V_{0}$ does not impact the odds ratio but the fixed value of $M_{0}$ does.

Supplemental Table 2B. Comparison of rate ratio of NTM infection under

different Molybdenum and Vanadium scenarios in a Medicare population.

| Midwest | 25% Quantile Molybdenum (0.826 μg/L) | 50% quantile Molybdenum (1.465 μg/L) | 75% Quantile Molybdenum (3.184 μg/L) | 90% Quantile Molybdenum (6.012 μg/L) |
| --- | --- | --- | --- | --- |
| Vanadium rate ratio  95% CI  p-value | 1.126  (0.97, 1.30)  0.197 | 1.125  (1.00, 1.26)  0.044 | 1.125  (1.01, 1.26)  0.034 | 1.125  (0.95, 1.33)  0.360 |
| Northeast | 25% Quantile Molybdenum (0.707 μg/L) | 50% Quantile Molybdenum (0.707 μg/L) | 75% Quantile Molybdenum (0.811 μg/L) | 90% Quantile Molybdenum (1.042 μg/L) |
| Vanadium rate ratio  95% CI  p-value | 0.946  (0.57, 1.57)  1.000 | 0.946  (0.57, 1.57)  1.000 | 1.010  (0.64, 1.60)  1.000 | 1.155  (0.74, 1.81)  0.976 |
| South | 25% Quantile Molybdenum (0.707 μg/L) | 50% Quantile Molybdenum (0.707 μg/L) | 75% Quantile Molybdenum (0.978 μg/L) | 90% Quantile Molybdenum (1.892 μg/L) |
| Vanadium rate ratio  95% CI  p-value | 1.183  (1.07, 1.30)  1.84x10^-5^ | 1.183  (1.07, 1.30)  1.36x10^-5^ | 1.151  (1.05, 1.26)  1.23x10^-4^ | 1.070  (0.99, 1.16)  0.169 |
| West | 25% Quantile Molybdenum (0.709 μg/L) | 50% Quantile Molybdenum (1.141 μg/L) | 75% Quantile Molybdenum (2.065 μg/L) | 90% Quantile Molybdenum (3.941 μg/L) |
| Vanadium rate ratio  95% CI  p-value | 1.149  (1.03, 1.29)  6.28x10^-3^ | 1.119  (1.02, 1.23)  0.012 | 1.074  (0.97, 1.19)  0.330 | 1.016  (0.88, 1.18)  1.000 |

The rate ratio compares the rate of NTM infection when Vanadium increases from a fixed value, $V_{0}$, to $V_{0}+1$ while Molybdenum is held fixed at a value, $M_{0}$. We computed the rate ratios under four fixed values of $M_{0}$. The fixed value of $V_{0}$ does not impact the rate ratio but the fixed value of $M_{0}$ does.

Supplemental Table 3A. Full Model Output - CF Trace Metal Model

| Term | Estimate | STD.Error | Statistic | P.Value |
| --- | --- | --- | --- | --- |
| (Intercept) | -2.707 | 0.456 | -5.940 | 2.85e-09 |
| Chromium | 0.055 | 0.066 | 0.839 | 4.02e-01 |
| Molybdenum | -0.061 | 0.050 | -1.224 | 2.21e-01 |
| Vanadium | 0.276 | 0.117 | 2.364 | 1.81e-02 |
| Cobalt | -0.022 | 0.094 | -0.232 | 8.17e-01 |
| Strontium | 0.151 | 0.076 | 1.977 | 4.80e-02 |
| Study_RegionNortheastern | -0.160 | 0.885 | -0.181 | 8.56e-01 |
| Study_RegionSouthern | 1.301 | 0.536 | 2.428 | 1.52e-02 |
| Study_RegionWestern | 0.839 | 0.561 | 1.494 | 1.35e-01 |
| year_rain | 0.026 | 0.011 | 2.309 | 2.09e-02 |
| FL | 0.000 | 0.001 | 0.291 | 7.71e-01 |
| waterSW | -0.293 | 0.150 | -1.951 | 5.11e-02 |
| waterMX | -0.020 | 0.130 | -0.152 | 8.79e-01 |
| age | 0.010 | 0.003 | 3.075 | 2.11e-03 |
| Gender | -0.038 | 0.085 | -0.452 | 6.51e-01 |
| MedIncome | 0.005 | 0.005 | 1.043 | 2.97e-01 |
| CLGA | 0.092 | 0.126 | 0.732 | 4.64e-01 |
| Chlo | 0.022 | 0.105 | 0.208 | 8.36e-01 |
| Hypo | 0.058 | 0.107 | 0.544 | 5.86e-01 |
| Other | 0.249 | 0.102 | 2.435 | 1.49e-02 |
| Chromium:Study_RegionNortheastern | 0.230 | 0.204 | 1.132 | 2.58e-01 |
| Chromium:Study_RegionSouthern | -0.138 | 0.087 | -1.595 | 1.11e-01 |
| Chromium:Study_RegionWestern | -0.015 | 0.083 | -0.176 | 8.60e-01 |
| Study_RegionNortheastern:Molybdenum | 0.103 | 0.239 | 0.431 | 6.67e-01 |
| Study_RegionSouthern:Molybdenum | -0.079 | 0.078 | -1.010 | 3.12e-01 |
| Study_RegionWestern:Molybdenum | 0.332 | 0.120 | 2.755 | 5.87e-03 |
| Study_RegionNortheastern:Vanadium | 0.366 | 0.326 | 1.120 | 2.63e-01 |
| Study_RegionSouthern:Vanadium | -0.058 | 0.138 | -0.421 | 6.74e-01 |
| Study_RegionWestern:Vanadium | -0.283 | 0.132 | -2.144 | 3.20e-02 |
| Study_RegionNortheastern:Cobalt | -0.009 | 0.126 | -0.068 | 9.46e-01 |
| Study_RegionSouthern:Cobalt | -0.015 | 0.101 | -0.145 | 8.85e-01 |
| Study_RegionWestern:Cobalt | -0.355 | 0.281 | -1.263 | 2.06e-01 |
| Study_RegionNortheastern:Strontium | -0.231 | 0.128 | -1.801 | 7.17e-02 |
| Study_RegionSouthern:Strontium | -0.136 | 0.086 | -1.579 | 1.14e-01 |
| Study_RegionWestern:Strontium | -0.138 | 0.124 | -1.111 | 2.66e-01 |
| Molybdenum:Vanadium | -0.120 | 0.080 | -1.510 | 1.31e-01 |
| year_rain:FL | 0.000 | 0.000 | -0.189 | 8.50e-01 |
| Study_RegionNortheastern:year_rain | 0.002 | 0.023 | 0.076 | 9.39e-01 |
| Study_RegionSouthern:year_rain | -0.017 | 0.013 | -1.363 | 1.73e-01 |
| Study_RegionWestern:year_rain | -0.024 | 0.013 | -1.815 | 6.95e-02 |
| Study_RegionNortheastern:FL | 0.006 | 0.004 | 1.519 | 1.29e-01 |
| Study_RegionSouthern:FL | 0.002 | 0.001 | 1.126 | 2.60e-01 |
| Study_RegionWestern:FL | 0.001 | 0.002 | 0.673 | 5.01e-01 |
| Study_RegionNortheastern:waterSW | 0.886 | 0.289 | 3.065 | 2.18e-03 |
| Study_RegionSouthern:waterSW | 0.048 | 0.179 | 0.269 | 7.88e-01 |
| Study_RegionWestern:waterSW | 0.584 | 0.263 | 2.217 | 2.66e-02 |
| Study_RegionNortheastern:waterMX | 0.352 | 0.275 | 1.282 | 2.00e-01 |
| Study_RegionSouthern:waterMX | -0.166 | 0.162 | -1.027 | 3.05e-01 |
| Study_RegionWestern:waterMX | -0.132 | 0.206 | -0.643 | 5.20e-01 |
| Study_RegionNortheastern:age | 0.006 | 0.005 | 1.317 | 1.88e-01 |
| Study_RegionSouthern:age | 0.002 | 0.004 | 0.400 | 6.89e-01 |
| Study_RegionWestern:age | 0.001 | 0.005 | 0.213 | 8.32e-01 |
| Study_RegionNortheastern:Gender | 0.166 | 0.123 | 1.353 | 1.76e-01 |
| Study_RegionSouthern:Gender | 0.010 | 0.105 | 0.097 | 9.23e-01 |
| Study_RegionWestern:Gender | 0.165 | 0.116 | 1.432 | 1.52e-01 |
| Study_RegionNortheastern:MedIncome | -0.010 | 0.006 | -1.619 | 1.06e-01 |
| Study_RegionSouthern:MedIncome | -0.003 | 0.005 | -0.682 | 4.95e-01 |
| Study_RegionWestern:MedIncome | 0.006 | 0.006 | 0.957 | 3.39e-01 |
| Study_RegionNortheastern:CLGA | -0.141 | 0.176 | -0.799 | 4.24e-01 |
| Study_RegionSouthern:CLGA | -0.158 | 0.145 | -1.084 | 2.78e-01 |
| Study_RegionWestern:CLGA | -0.274 | 0.161 | -1.695 | 9.01e-02 |
| Study_RegionNortheastern:Chlo | 0.352 | 0.171 | 2.063 | 3.92e-02 |
| Study_RegionSouthern:Chlo | 0.279 | 0.128 | 2.186 | 2.88e-02 |
| Study_RegionWestern:Chlo | -0.110 | 0.154 | -0.715 | 4.74e-01 |
| Study_RegionNortheastern:Hypo | 0.201 | 0.202 | 0.999 | 3.18e-01 |
| Study_RegionSouthern:Hypo | -0.045 | 0.127 | -0.352 | 7.25e-01 |
| Study_RegionWestern:Hypo | 0.001 | 0.214 | 0.002 | 9.98e-01 |
| Study_RegionNortheastern:Other | -0.274 | 0.156 | -1.757 | 7.90e-02 |
| Study_RegionSouthern:Other | -0.358 | 0.125 | -2.864 | 4.18e-03 |
| Study_RegionWestern:Other | -0.135 | 0.140 | -0.961 | 3.36e-01 |
| Study_RegionNortheastern:Molybdenum:Vanadium | 0.840 | 0.560 | 1.500 | 1.34e-01 |
| Study_RegionSouthern:Molybdenum:Vanadium | 0.017 | 0.096 | 0.181 | 8.56e-01 |
| Study_RegionWestern:Molybdenum:Vanadium | 0.130 | 0.094 | 1.381 | 1.67e-01 |
| Study_RegionNortheastern:year_rain:FL | 0.000 | 0.000 | -1.801 | 7.18e-02 |
| Study_RegionSouthern:year_rain:FL | 0.000 | 0.000 | -1.085 | 2.78e-01 |
| Study_RegionWestern:year_rain:FL | 0.000 | 0.000 | -0.989 | 3.23e-01 |

Supplemental Table 3B. Full Model Output - Medicare Trace Metal Model

| Term | Estimate | STD.Error | Statistic | P.Value |
| --- | --- | --- | --- | --- |
| (Intercept) | -9.207 | 0.861 | -10.693 | 1.10e-26 |
| Chromium | -0.029 | 0.028 | -1.055 | 2.91e-01 |
| Molybdenum | -0.035 | 0.022 | -1.579 | 1.14e-01 |
| Vanadium | 0.118 | 0.044 | 2.701 | 6.90e-03 |
| Cobalt | 0.046 | 0.038 | 1.208 | 2.27e-01 |
| Strontium | -0.039 | 0.032 | -1.212 | 2.25e-01 |
| waterSW | -0.016 | 0.062 | -0.262 | 7.93e-01 |
| waterMX | -0.036 | 0.059 | -0.612 | 5.41e-01 |
| year_rain | 0.015 | 0.005 | 2.877 | 4.02e-03 |
| FL | 0.000 | 0.000 | -0.731 | 4.65e-01 |
| CLGA | 0.161 | 0.051 | 3.167 | 1.54e-03 |
| Chlo | 0.277 | 0.060 | 4.652 | 3.29e-06 |
| Hypo | 0.071 | 0.053 | 1.324 | 1.85e-01 |
| Other | -0.140 | 0.153 | -0.915 | 3.60e-01 |
| age_po75 | 0.018 | 0.008 | 2.281 | 2.25e-02 |
| sex_pf | 0.016 | 0.016 | 0.977 | 3.29e-01 |
| MedIncome | 0.008 | 0.002 | 4.890 | 1.01e-06 |
| Study_RegionNortheastern | -5.780 | 1.593 | -3.628 | 2.85e-04 |
| Study_RegionSouthern | 2.145 | 1.014 | 2.115 | 3.44e-02 |
| Study_RegionWestern | 5.404 | 1.235 | 4.377 | 1.20e-05 |
| Chromium:Study_RegionNortheastern | 0.065 | 0.163 | 0.396 | 6.92e-01 |
| Chromium:Study_RegionSouthern | -0.067 | 0.038 | -1.783 | 7.46e-02 |
| Chromium:Study_RegionWestern | 0.051 | 0.040 | 1.258 | 2.08e-01 |
| Study_RegionNortheastern:Molybdenum | 0.249 | 0.160 | 1.554 | 1.20e-01 |
| Study_RegionSouthern:Molybdenum | 0.079 | 0.035 | 2.243 | 2.49e-02 |
| Study_RegionWestern:Molybdenum | 0.204 | 0.062 | 3.315 | 9.15e-04 |
| Study_RegionNortheastern:Vanadium | 0.146 | 0.194 | 0.752 | 4.52e-01 |
| Study_RegionSouthern:Vanadium | -0.004 | 0.053 | -0.084 | 9.33e-01 |
| Study_RegionWestern:Vanadium | -0.012 | 0.055 | -0.223 | 8.24e-01 |
| Molybdenum:Vanadium | 0.000 | 0.029 | -0.010 | 9.92e-01 |
| Study_RegionNortheastern:Cobalt | -0.065 | 0.057 | -1.137 | 2.56e-01 |
| Study_RegionSouthern:Cobalt | -0.057 | 0.040 | -1.439 | 1.50e-01 |
| Study_RegionWestern:Cobalt | -0.215 | 0.119 | -1.807 | 7.08e-02 |
| Study_RegionNortheastern:Strontium | 0.046 | 0.069 | 0.666 | 5.06e-01 |
| Study_RegionSouthern:Strontium | -0.008 | 0.038 | -0.206 | 8.37e-01 |
| Study_RegionWestern:Strontium | 0.023 | 0.059 | 0.398 | 6.91e-01 |
| Study_RegionNortheastern:waterSW | 0.204 | 0.138 | 1.479 | 1.39e-01 |
| Study_RegionSouthern:waterSW | -0.213 | 0.075 | -2.833 | 4.61e-03 |
| Study_RegionWestern:waterSW | 0.187 | 0.125 | 1.499 | 1.34e-01 |
| Study_RegionNortheastern:waterMX | -0.001 | 0.136 | -0.009 | 9.93e-01 |
| Study_RegionSouthern:waterMX | -0.087 | 0.074 | -1.177 | 2.39e-01 |
| Study_RegionWestern:waterMX | 0.131 | 0.105 | 1.250 | 2.11e-01 |
| year_rain:FL | 0.000 | 0.000 | 0.967 | 3.33e-01 |
| Study_RegionNortheastern:year_rain | -0.012 | 0.015 | -0.773 | 4.39e-01 |
| Study_RegionSouthern:year_rain | 0.000 | 0.006 | -0.084 | 9.33e-01 |
| Study_RegionWestern:year_rain | -0.018 | 0.006 | -3.106 | 1.89e-03 |
| Study_RegionNortheastern:FL | 0.002 | 0.003 | 0.589 | 5.56e-01 |
| Study_RegionSouthern:FL | 0.001 | 0.001 | 2.040 | 4.13e-02 |
| Study_RegionWestern:FL | -0.001 | 0.001 | -1.246 | 2.13e-01 |
| Study_RegionNortheastern:CLGA | -0.250 | 0.092 | -2.710 | 6.74e-03 |
| Study_RegionSouthern:CLGA | -0.223 | 0.061 | -3.667 | 2.45e-04 |
| Study_RegionWestern:CLGA | -0.100 | 0.085 | -1.177 | 2.39e-01 |
| Study_RegionNortheastern:Chlo | -0.188 | 0.114 | -1.659 | 9.72e-02 |
| Study_RegionSouthern:Chlo | 0.055 | 0.073 | 0.752 | 4.52e-01 |
| Study_RegionWestern:Chlo | -0.153 | 0.105 | -1.457 | 1.45e-01 |
| Study_RegionNortheastern:Hypo | 0.035 | 0.094 | 0.375 | 7.08e-01 |
| Study_RegionSouthern:Hypo | 0.017 | 0.065 | 0.254 | 7.99e-01 |
| Study_RegionWestern:Hypo | 0.148 | 0.088 | 1.683 | 9.23e-02 |
| Study_RegionNortheastern:Other | -0.032 | 0.228 | -0.138 | 8.90e-01 |
| Study_RegionSouthern:Other | 0.251 | 0.199 | 1.259 | 2.08e-01 |
| Study_RegionWestern:Other | 0.118 | 0.211 | 0.560 | 5.75e-01 |
| Study_RegionNortheastern:age_po75 | -0.077 | 0.016 | -4.898 | 9.68e-07 |
| Study_RegionSouthern:age_po75 | -0.005 | 0.010 | -0.503 | 6.15e-01 |
| Study_RegionWestern:age_po75 | 0.021 | 0.014 | 1.501 | 1.33e-01 |
| Study_RegionNortheastern:sex_pf | 0.179 | 0.034 | 5.308 | 1.11e-07 |
| Study_RegionSouthern:sex_pf | -0.021 | 0.019 | -1.070 | 2.85e-01 |
| Study_RegionWestern:sex_pf | -0.119 | 0.028 | -4.313 | 1.61e-05 |
| Study_RegionNortheastern:MedIncome | -0.002 | 0.002 | -0.645 | 5.19e-01 |
| Study_RegionSouthern:MedIncome | -0.003 | 0.002 | -1.373 | 1.70e-01 |
| Study_RegionWestern:MedIncome | 0.004 | 0.002 | 1.677 | 9.35e-02 |
| Study_RegionNortheastern:Molybdenum:Vanadium | 0.527 | 0.329 | 1.605 | 1.09e-01 |
| Study_RegionSouthern:Molybdenum:Vanadium | -0.090 | 0.034 | -2.626 | 8.65e-03 |
| Study_RegionWestern:Molybdenum:Vanadium | -0.054 | 0.040 | -1.357 | 1.75e-01 |
| Study_RegionNortheastern:year_rain:FL | 0.000 | 0.000 | -0.547 | 5.84e-01 |
| Study_RegionSouthern:year_rain:FL | 0.000 | 0.000 | -2.070 | 3.84e-02 |
| Study_RegionWestern:year_rain:FL | 0.000 | 0.000 | 0.473 | 6.37e-01 |

Supplemental Table 4.

A) The odds ratio of MAC PI among persons with cystic fibrosis in the United States for trace metals and water disinfectants.

B) The odds ratio of *M. abscessus* PI among persons with cystic fibrosis in the United States for trace metals.

Each row indicates the estimated odds ratio, the 95% confidence interval, and the significance level of each odds ratio.

| Region | 1. MAC PI among CF patients^1^   OR (95% CI) p-value^2^ | 1. *M. abscessus* PI among CF patients^1^   OR (95% CI) p-value^2^ |
| --- | --- | --- |
| Midwest | Chromium 1.12 (0.90,1.38) 0.590  Cobalt 0.99 (0.74,1.33) 0.999  Molybdenum 0.99 (0.85,1.16) 0.999  Strontium 1.21 (0.94,1.56) 0.229  **Vanadium 1.36 (1.01,1.83) 0.043**  Molybdenum:Vanadium 0.87 (0.68,1.12) 0.430 | Chromium 0.99 (0.72,1.38) 0.999  Cobalt 0.92 (0.51,1.66) 0.995  Molybdenum 0.93 (0.74,1.18) 0.899  Strontium 1.22 (0.85,1.74) 0.546  Vanadium 1.12 (0.70,1.80) 0.961  Molybdenum:Vanadium 0.87 (0.57,1.30) 0.999 |
| Northeast | Chromium 1.09 (0.60,1.96) 0.994  Cobalt 0.70 (0.49,0.98) 0.035  Molybdenum 0.39 (0.14,1.11) 0.095  Strontium 1.02 (0.74,1.41) 0.999  Vanadium 1.47 (0.74,2.91) 0.497  Molybdenum:Vanadium 5.93 (0.92,38.5) 0.069 | Chromium 1.92 (0.73,5.04) 0.323  Cobalt 1.12 (0.79,1.58) 0.889  Molybdenum 1.35 (0.56,3.27) 0.867  Strontium 0.93 (0.57,1.52) 0.993  Vanadium 1.07 (0.37,3.06) 0.999  Molybdenum:Vanadium 0.34 (0.02,4.87) 0.775 |
| South | Chromium 0.94 (0.78,1.14) 0.907  Cobalt 0.98 (0.87,1.09) 0.975  Molybdenum 0.87 (0.70,1.08) 0.351  Strontium 0.96 (0.84,1.09) 0.869  Vanadium 1.23 (0.94,1.60) 0.208  Molybdenum:Vanadium 0.94 (0.78,1.13) 0.863 | Chromium 0.91 (0.74,1.12) 0.677  Cobalt 0.98 (0.85,1.12) 0.986  Molybdenum 0.89 (0.70,1.13) 0.629  Strontium 0.99 (0.85,1.15) 0.999  **Vanadium 1.36 (1.03,1.80) 0.025**  Molybdenum:Vanadium 0.88 (0.71,1.08) 0.407 |
| West | Chromium 1.02 (0.86,1.21) 0.998  Cobalt 0.77 (0.34,1.73) 0.891  **Molybdenum 1.39 (1.08,1.80) 0.005**  Strontium 0.97 (0.71,1.34) 0.999  Vanadium 0.95 (0.76,1.17) 0.949  Molybdenum:Vanadium 0.99 (0.84,1.18) 0.999 | Chromium 1.07 (0.85,1.35) 0.926  Cobalt 0.75 (0.23,2.48) 0.961  Molybdenum 1.35 (0.96,1.89) 0.113  Strontium 0.94 (0.61,1.44) 0.992  Vanadium 0.97 (0.73,1.28) 0.997  Molybdenum:Vanadium 1.01 (0.81,1.27) 0.999 |

^1^Source water type, sociodemographic variables, climate variables, water disinfectants, and interaction term (between precipitation and floods) are also controlled for each logistic model.

^2^Confidence intervals and p-values are adjusted for multiple comparisons.

Molybdenum:Vanadium indicates an interaction term.

Supplemental Table 5.

A) The odds ratio of NTM PI among persons with cystic fibrosis in the United States for trace metals.

B) The rate ratio of NTM PI among Medicare recipients in the United States for trace metals.

Each row indicates the estimated effect estimate (odds ratio or rate ratio), the 95% confidence interval for the effect estimate, and the significance level of the effect estimate associated with a specific covariate.

Water disinfectants are excluded from this analysis.

| Region | 1. NTM PI among CF patients   OR (95% CI) p-value | 1. NTM PI among Medicare recipients   RR (95% CI) p-value |
| --- | --- | --- |
| Midwest | Chromium 1.06 (0.90,1.24) 0.872  Cobalt 0.98 (0.77,1.24) 0.999  Molybdenum 0.95 (0.85,1.08) 0.859  Strontium 1.16 (0.96,1.41) 0.179  Vanadium 1.24 (0.98,1.57) 0.087  Molybdenum:Vanadium 0.89 (0.73,1.08) 0.430 | Chromium 0.97 (0.91,1.05) 0.919  Cobalt 1.05 (0.95,1.16) 0.582  Molybdenum 0.95 (0.90,1.01) 0.150  Strontium 0.96 (0.88,1.04) 0.609  Vanadium 1.09 (0.99,1.20) 0.079  Molybdenum:Vanadium 0.99 (0.93,1.08) 1.000 |
| Northeast | Chromium 1.33 (0.82,2.15) 0.448  Cobalt 0.97 (0.79,1.19) 0.993  Molybdenum 0.77 (0.40,1.49) 0.796  Strontium 0.92 (0.72,1.19) 0.901  Vanadium 1.36 (0.79,2.33) 0.509  Molybdenum:Vanadium 2.05 (0.52,8.17) 0.578 | Chromium 1.05 (0.71,1.56) 0.995  Cobalt 0.98 (0.88,1.09) 0.969  Molybdenum 0.99 (0.71,1.40) 0.999  Strontium 0.99 (0.86,1.16) 0.999  Vanadium 1.01 (0.67,1.52) 0.999  Molybdenum:Vanadium 1.67 (0.72,3.90) 0.418 |
| South | Chromium 0.92 (0.80,1.06) 0.456  Cobalt 0.97 (0.88,1.05) 0.761  Molybdenum 0.89 (0.76,1.03) 0.145  Strontium 1.02 (0.92,1.12) 0.992  **Vanadium 1.27 (1.05,1.54) 8.7x10^-43^**  Molybdenum:Vanadium 0.90 (0.79,1.03) 0.194 | **Chromium 0.89 (0.84,0.96) 1.7x10^-4^**  Cobalt 0.99 (0.96,1.02) 0.923  **Molybdenum 1.08 (1.01,1.16) 0.022**  Strontium 0.97 (0.92,1.02) 0.372  **Vanadium 1.16 (1.07,1.26) 3.0x10^-8^**  Molybdenum:Vanadium 0.89 (0.85,0.94) 0.430 |
| West | Chromium 1.04 (0.92,1.18) 0.894  Cobalt 0.69 (0.36,1.33) 0.489  **Molybdenum 1.33 (1.09,1.61) 0.001**  Strontium 1.01 (0.79,1.29) 0.999  Vanadium 0.99 (0.85,1.17) 0.999  Molybdenum:Vanadium 1.01 (0.89,1.15) 0.999 | Chromium 1.05 (0.98,1.13) 0.307  Cobalt 0.90 (0.68,1.19) 0.814  **Molybdenum 1.13 (1.01,1.26) 0.021**  Strontium 0.99 (0.88,1.13) 0.999  Vanadium 1.09 (0.99,1.19) 0.063  Molybdenum:Vanadium 0.95 (0.88,1.02) 0.253 |

Source water type, sociodemographic variables, climate variables and interaction terms (between precipitation and floods) are also controlled for in the logistic model (A) and the negative binomial model (B).
